# Brain Injury and Severe Eating Difficulties at Admission—Patient Perspective Nine to Fifteen Months after Discharge: A Pilot Study

**DOI:** 10.3390/brainsci7080096

**Published:** 2017-08-07

**Authors:** Annette Kjaersgaard, Hanne Kaae Kristensen

**Affiliations:** 1 Hammel Neurorehabilitation Centre and University Research Clinic, Aarhus University, 8450 Hammel, Denmark; 2 Institute of Clinical Research, University of Southern Denmark and Health Sciences Research Centre, University College Lillebaelt, 5230 Odense M, Denmark; hkkr@ucl.dk

**Keywords:** dysphagia, neurorehabilitation, qualitative interview

## Abstract

The purpose of this pilot study was to explore and interpret the way that individuals with acquired brain injury, admitted to inpatient neurorehabilitation with severe eating difficulties, experienced eating nine to fifteen months after discharge. Four individuals with acquired brain injury were interviewed via qualitative semi-structured interviews. An explorative study was conducted to study eating difficulties. Qualitative content analysis was used. Four main themes emerged from the analysis: personal values related to eating, swallowing difficulties, eating and drinking, meals and social life. Three predominating experiences were: fed by tube, “relearning” to eat, and eating meals together. The preliminary results regarding the four participants suggest that the meaning of food and being able to eat and take part in meals may be nearly the same as before the injury; however, having the ability to eat reduced or lost completely, even temporarily, was unexpected and difficult, and caused strong emotional reactions, even 18 months after injury. Time spent using a feeding tube had a negative, but not persistent, impact on quality-of-life. The preliminary findings provide knowledge regarding the patient perspective of adapting to and developing new strategies for activities related to eating, however, further prospective, longitudinal research in a larger scale and with repeated interviews is needed.

## 1. Introduction

Stroke and traumatic brain injury (TBI) are the main causes of acquired brain injury (ABI). In 2009, there were an estimated 12,500 cases of hospitalisations from stroke and an estimated 9500 cases of hospitalisation from TBI in Denmark [1]. The incidence of dysphagia is reported as from 27% to 93% among patients with ABI in neurorehabilitation [2,3].

Living with eating difficulties after ABI involves a complex and difficult process of adjusting to a new way of eating, as well as losses involving mealtime activities [4]. Little is known about eating difficulties and adaptation to lasting eating difficulties after discharge from inpatient neurorehabilitation [5].

In this study, eating difficulties are defined as difficulties with ingestion, swallowing, eating and drinking, using the subcategories outlined in the International Classification of Functioning, Disability and Health [6]. Eating difficulties may cause dehydration, malnutrition, aspiration and pneumonia, and can contribute to a less optimal and increased duration of rehabilitation, leading to feelings of shame, dependency and other negative experiences [4,7].

Eating difficulties have profound effects on individuals [8] and living with eating difficulties is a complex phenomenon [4] that is common in individuals recovering from stroke and living in the community [9]. Eating difficulties can impact social opportunities and the pleasure derived from meals, as well as the quality of social relationships for the person with ABI, undermining their health and confidence. Individuals with eating difficulties may become isolated, feel excluded by others, and be anxious and distressed at mealtime [10]; they often experience considerable limitations in their everyday life. Medin et al. [11] described the complexity of experiencing eating difficulties after a stroke, and issues related to feeling in control as being based on a number of strategies: being careful when eating, avoiding social activities, needing help from others, analysing the consequences of eating different foods, and eating safely and properly.

Research studies often focus on improving the physiological evaluation and treatment of dysphagia and underemphasise the evaluation and management of other factors, such as the effect on a person’s eating abilities [12]. There are questions to be answered about how patients with ABI perceive and adjust to changes in everyday activities related to eating [13] after discharge from neurorehabilitation. Important knowledge must be acquired by the patient with ABI and eating difficulties, their relatives, and the multidisciplinary rehabilitation team when describing common goal setting and treatment planning during inpatient neurorehabilitation.

### Theoretical Framework

The concept of adaptation involves a way of thinking about what happens when a disability changes the conditions for an individual’s daily occupation [14]. The concept of adaptation is essential within the context of rehabilitation, and is used with various definitions [13]. In this study, adaptation was defined as “the process by which a person maintained a useful relationship to the environment” [15]. The focus on adaptation in this study was mainly at the intrapersonal level [16,17,18], in which adaptation to an ever-changing environment while experiencing the challenges of a loss of abilities is complex, and can be a long process, particularly when adapting to major changes in one’s life [19]. The current pilot study therefore aims to explore and interpret how individuals with ABI, admitted to inpatient neurorehabilitation with severe eating difficulties, experienced eating nine to fifteen months after discharge.

## 2. Materials and Methods

This pilot study forms one component of a mixed methods investigation of difficulties with swallowing and eating following ABI. The first phase was a prospective randomised controlled trial (RCT) of assessment involving facial-oral tract therapy versus fiberoptic endoscopic evaluation of swallowing during inpatient neurorehabilitation; we compared the risk of aspiration pneumonia in patients with ABI [20] and the time to initiation of oral intake and recovery of total oral intake before discharge [21]. The second phase, which is the focus of this paper, was a preliminary, explorative, qualitative interview study conducted to gather the patient perspective of eating nine to fifteen months after discharge, and to refine the methodology for a later longitudinal study.

### 2.1. Sampling and Participants

The participants were recruited through a criterion sampling strategy [22]. The inclusion criteria for this pilot study were: (1) diagnosed with an ABI and enrolled in the study mentioned below, (2) severe dysphagia at the time of admission to inpatient neurorehabilitation (Functional Independence Measure (FIM) score of 1 for the item “Eating”), (3) have or have had a feeding tube, and (4) be able to understand the interview questions and express/describe their experience in Danish (FIM score 5–7 for items “Expression” and “Memory” at the time of discharge from neurorehabilitation). Four participants were retrospectively selected, with help from local clinical dysphagia experts, from participants involved in the RCT [20]—a total of 119 patients with ABI. There were no withdrawals from the study. See Table 1 for further clinical characteristics.

### 2.2. Ethics

The pilot study was performed according to the Helsinki Declaration [23]. The Danish Data Protection Agency was notified and the collection of data was handled according to their guidelines (Journal No. 2007-58-0010). According to Danish legislation, only biomedical trials require approval by a Research Ethics Committee, and therefore, this study did not require such approval. The participants gave their verbal and written informed consent for participation and were guaranteed confidentiality. Participation was voluntary, and the participants could withdraw from the study at any time. Interviewing people following ABI, when they are experiencing a loss of bodily and cognitive functions, presents unique challenges [24] requiring sensitivity and tactfulness. The first author had extensive experience treating persons with severe ABI and was familiar with the challenges in obtaining the full and active participation of a person with ABI.

### 2.3. Data Collection

The quantitative data (primary data set from the RCT [20]) was collected from June 2009 to April 2010, and the qualitative data was collected in December 2010 and was timed and directed according to the progress of the RCT. The quantitative data set, which was documented from the medical records by the treating occupational therapist, was used to describe patient characteristics and to contextualise the participants in this pilot study (see Table 1). The FIM [25] was rated by the multidisciplinary team, as standard practice at the clinic, which includes at the time of admission, every fourth week during neurorehabilitation, and at discharge.

The first author initiated the qualitative interviews after initial contact two months prior to the study. At that point, the participants received an information letter with the informed consent form. On the day of the interview, the participants had the opportunity to ask questions about the study and complete the informed consent forms.

The empirical data was collected using semi-structured interviews. An interview guide was used during the interviews. The semi-structured interview guide (Table 2) consisted of topics derived from the literature [26]. The questions were open-ended [27] and were developed after a pilot interview undertaken by the first author with one person with ABI under the supervision of a senior researcher, during which the terminology was simplified and prompts were identified.

The participants were interviewed once in their own homes between nine and eighteen months after their injury, in their semi-stable phase after discharge from inpatient neurorehabilitation where they were going on with life [28].

During the interviews, the participants were encouraged to give examples and describe actual situations they had experienced [27], and they were asked to describe their present and previous experiences and management of eating difficulties.

The first author performed all the interviews. The interviews, which lasted between 30 and 60 min, were tape-recorded and fully transcribed verbatim by a person not involved in the study. The participants were sent transcripts of the interviews in order to invite the participants to comment on the transcriptions. The transcribed data were then de-identified and pseudonym names were created.

### 2.4. Data Analysis

The interviews were analysed using content analysis [29]. Electronic transcripts of the four interviews were reviewed. The authors carried out the content analysis. The focus of the data analysis was the subjective individual experiences. As described by Elo and Kyngäs [30], content analysis is a systematic method of delineating observable meanings (referred to herein as codes) in data and classifying them into categories that exemplify a phenomenon (termed themes in this study).

As recommended by Graneheim and Lundman [29], the researchers employed abstraction of the data—an approach used to interpret the data in sequential units of codes, categories, sub-categories and themes. The transcripts were read separately, thoroughly and as a whole several times by the first author to obtain an overall understanding of each person and the data. The text was extracted according to meaning units. The first author condensed and coded the meaning units. The coding process was performed using the freeware computer programme Open Code [31]. Both authors compared the codes in each interview to identify similarities and differences, and subsequently, codes that reflected similar aspects were grouped into units of similar meanings, before ultimately being divided into categories and sub-categories. During the process, which involved going back and forth between the text (meaning units) and the emerging categories to ensure internal validity, the authors continuously discussed and reached a consensus on the final categories and sub-categories. See Table 3 for an example of the codes, sub-categories, categories and themes from the content analysis.

Validation in qualitative research is a process of continually checking, questioning and theoretically interpreting the findings [32] in order to secure trustworthiness [29]. The step for improving credibility included careful efforts to ensure that the speech of the participants was properly understood. After transcription of the interview, the first author returned the transcripts to the participants to verify that the researcher had understood their statements correctly. The researcher triangulation undertaken by two skilled researchers involved extracting meaning units, formulating themes and interpreting the data and use of theories of adaptation to discuss the findings. The triangulation was performed to contribute to the credibility of the study.

## 3. Results

The analysis generated the following four main themes about the patient perspective of: “personal values related to eating”, “swallowing difficulties”, “eating and drinking” and “meals and social life” (Table 4). The results first describe how the four patients experienced these themes, and then how they adjusted to the environmental challenges after discharge.

The following short personal presentation describes each of the four participants (Table 1), followed by a detailed description of their experiences of eating abilities following ABI.

- Marie (18 years old) was unemployed before the ABI, and nine months after the injury, she was a dedicated student and lived with her father, younger brother and their dog. She spent a significant amount of time at home with her brother or concentrating on her homework and on seeing friends.
- Anne (27 years old) worked as a dental assistant before the ABI, and 18 months after the injury, she worked three hours a day in her previous job and was an active mother with two small children and a self-employed husband. The family lived in a large newly-built house with no practical help. She spent most of her spare time at home, but once a week, she engaged in sports.
- Hans (30 years old) worked as a craftsman before the ABI and operated a small business in addition to his work as a craftsman. He was still operating his small business 16 months after the injury, but no longer worked as a craftsman. He lived alone in his own house and had little practical help. His hobby was also his work. He was in close contact with his parents, who lived nearby with his younger brother, whom he often helped, and with many old friends.
- Peter (60 years old) worked as a caretaker before the ABI and was a creative handyman who loved to rebuild the family house. Sixteen months after the injury, he did not work and spent most of his time at home alone or with his wife, who worked full time.

### 3.1. Personal Values Related to Eating

Two sub-categories were identified as describing the psychological conditions and experiences related to eating after injury.

#### 3.1.1. How to Swallow and Eat

Marie, Anne and Hans could swallow and eat as well as they could before the injury; there were no problems and no need for further rehabilitation after discharge. Peter described his swallowing difficulties, “I have gotten used to it. Of course, it is different than before, but when I think of it, I do not find it bad.” He felt the swallowing problems came on top of other problems and that he was “affected twice”, but now, “I feel I can eat most food […]. There is nothing to be worried about. I take things as they are.”

#### 3.1.2. Treatment Goals Concerning Swallowing and Eating

The participants described their treatment goals during neurorehabilitation very differently. Marie and Anne did not feel that they had ever had any swallowing and eating difficulties after the ABI. Hans said that he very quickly had a treatment goal, because, “On Christmas Eve I wanted duck for dinner—and duck I had!” However, being able to walk was his first priority—eating was a side issue. Peter’s main treatment goal was to be able to walk and then to use his left arm. Eating was his third priority.

### 3.2. Swallowing Difficulties

Three sub-categories were identified related to functional swallowing after injury.

#### 3.2.1. Symptoms like Choking, Coughing, Voice Clearing and Salivation

Marie, Anne and Hans did not remember any of these symptoms, but for Peter, swallowing caused these problems. He had a lot of saliva right after the injury and was both clearing his throat and coughing a lot:

[At that time, I got something stuck in my throat all the time, and when we had rye bread for dinner, it often got stuck in my throat, and I had to leave the table and drive out into the corridor, as the food was on the table].

At the time of the interview, Peter had only a minor problem with saliva when he was lying on his side in bed.

#### 3.2.2. Worries about Swallowing, Choking and Pneumonia

Marie and Anne did not have pneumonia during acute and inpatient neurorehabilitation. Hans had pneumonia in the acute phase, but said he was not afraid of aspiration or pneumonia. Marie noted “No one said that it could actually lead to pneumonia.” Peter had pneumonia during both acute and inpatient neurorehabilitation and said about pneumonia, “Yes, at the beginning I was [worried], but it stopped when I got home.”

#### 3.2.3. Hunger

None of the participants felt hunger in the period directly following the injury. Marie noted, “At that time, I was probably more shocked at everything that had happened, also because you are in a strange place and you just have to get used to being there. The last thing you think of is food.” She had no appetite and often felt that she ate only so as not to annoy the staff, “Although I did not feel like eating more, I then kept on eating.” Hans said, “I think it was the taste I lacked.” Peter did not think about food at first.

### 3.3. Eating and Drinking

Four sub-categories were identified related to the activity of eating (before and after injury).

#### 3.3.1. The Meaning of Food and Taking Part in Meals

Marie said, “I love really good food.” Anne described food as not meaning so much to her right after the injury, but at the time of the interview, she felt as she had before the injury and she loved new and exciting meals with friends and family. Peter also described himself as a lover of food, but this had changed since the injury, because now he had to consider his diabetes. When asked about the importance of food and the meaning of not being able to eat for a period of time, he answered:

[It does not matter. Maybe because I am a realist, and when I have been severely injured, it has these consequences, and then gradually, as time passes, I make progress.]

#### 3.3.2. Being Fed by a Tube

Right after the injury, both Marie and Anne had a nasal tube for about one month. Marie found it really annoying to have a tube in her nose, and it affected her mood. She remembered it was weird receiving water through the tube with a large syringe, and that it became quite cold in her stomach. She was happy the day it could be removed, although it was uncomfortable when it was pulled out. Anne did not remember the period with the nasal feeding tube. Hans had more than four months of tube feeding and “did not really think much about it. It was a necessary evil. It did not hurt.” He said it was a nuisance not being able to eat “because you never managed to taste the rubbish that came into the stomach directly.” To him, it had no negative impact.

[It was tiresome having to get up and lie flat on your bed in order to have something in your stomach. I was fed up with not having taste in my mouth. I actually remember it was easy and you could just lie down and be fed that way in order to think logically again.]

Peter was tube fed for more than five months and described it as a “terrible, horrible and tough time” receiving formula. He said he could not remember it “because I would rather not remember it. There are things you push into the background and bad experiences are such things I push away.” He remembered the equipment broke, but:

[... in its own way, formula is fine because I had something to eat that way, but I think it prolonged the time where I did not get solid food. I think it held me back a bit from—well I got formula—not starting to eat earlier.]

#### 3.3.3. Having the First Oral Intake after ABI

Marie remembered it was a little weird to chew and swallow again, and someone had to be with her when she ate in order for her to not aspirate or get anything stuck in her throat. Marie said:

[When I was not allowed to eat at all, I yearned for normal food, although I had been given enough. I just wanted to eat by myself. Just after I had the tube removed, I was used to getting enough to eat as the staff injected something into the tube, and suddenly there were trays with food, and I had lots of them during the day.]

Hans described his experience of having something in his mouth after a long period of tube feeding as, “It was great getting something that tasted of something in your mouth.”

#### 3.3.4. Food Tastes Differently

Marie and Peter said that their taste buds had changed since the injury. Peter had also stopped smoking and said food tasted much better now.

### 3.4. Meals and Social Life

One main sub-category was identified as related to participation in meals and social life.

#### Finding a Way with My Eating Difficulties

At the time of the interviews, three of the participants did not experience changes in their social lives caused by their eating difficulties. Hans noted that when his parents visited him during neurorehabilitation, they never ate in his presence because they felt sorry for him, “At that time, I never gave it a thought, but I think I would have found it a bit strange.” Peter remembered that at the beginning of the inpatient neurorehabilitation he had to eat in his room at the ward, with the staff watching him while eating and he had difficulty understanding why he could not eat with other patients, and was told “the reason was the other patients should not disturb him […]. The decision had been taken by others, and he just did so!” However, it was a little difficult for him to accept. Peter, who was the only participant who had changed his social life after discharge because of his eating difficulties, said his family did not frequent restaurants and “of course you miss it, but it is nothing important.”

[My focus is now on being rehabilitated so I can function reasonably at home. We often used to go on holiday. But not anymore; well, that is one of the consequences. I hope this will change when I have had further rehabilitation.]

## 4. Discussion

This was a small-scale pilot study to explore and interpret how individuals with ABI, admitted to inpatient neurorehabilitation with severe eating difficulties, experienced eating nine to fifteen months after discharge.

Overall, the preliminary results suggest that the meaning of food and being able to eat and take part in meals may be nearly the same as before the ABI; however, having the ability to eat reduced or lost completely, even temporarily, was unexpected and difficult, and caused strong emotional reactions, even 18 months after injury. The findings provide preliminary information on the patient perspective of adapting to and developing new strategies for activities related to eating.

In the following section, we highlight three predominant patient perspectives. In our interpretation and discussion of the findings regarding the processes of change over time, we used theories of adaptation to interpret experience and adjustment to a loss in functional skills for the individuals with impairment [16,17].

### 4.1. Fed by Tube

Tube feeding seemed to solve the participants’ swallowing and eating difficulties at the level of bodily functions and structure. As Hans remembered, “it was easy to lie down and be fed, to be able to think logically again”, however, the need for a feeding tube seemed to cause negative psychological experiences. Hans felt that he was “fed up not having taste in his mouth” and Peter described it as a “terrible, horrible and tough time receiving formula.” He said that he could not remember it because “he would rather not remember it.”

Especially for the two participants who had to use a Percutaneous Endoscopic Gastrostomy (PEG) long-term, the experiences were something they wanted to forget and Peter found that the feeding tube delayed his initiation of oral intake. Even the two participants needing a nasal feeding tube for a short time described their negative experiences, such as Marie, who remembered the nasal tube as being annoying and affecting her mood. The findings corresponded with a study by Rogers et al. [33], where persons with PEGs reported poor quality-of-life with significant deficits in all quality-of-life domains, such as family life, intimate relationships, social activities and hobbies, compared with persons not using PEGs.

The psychological experience of a period with a feeding tube had a negative, but not persistent, impact on the participants’ quality-of-life. After withdrawal of the feeding tube, the situation generally seemed to normalise, and each participant used different strategies to be able to participate in social gatherings. This included treating food and drink differently than they had before the injury, such as not taking liquid together with solid food or not going to restaurants. Although Peter did not return to the same social life as before the injury, he achieved an acceptable and meaningful new lifestyle, and the time when he required tube feeding seemed like “a closed chapter”. Our findings indicate tube feeding as having an important psychological factor for patients with ABI and a key focus area for the clinical awareness of health professionals in inpatient neurorehabilitation.

### 4.2. “Relearning” to Eat

During the time being fed with a tube, the participants yearned for normal food, and they often remembered their first oral intake. Doolittle [34] found that the first occasions when activities are resumed are important events: participants speak of the first time they accomplish something, such as the first cup of tea without thickener. The participants in this study had similar experiences, such as eating duck for Christmas.

Perry and McLaren [35] found that eating-related activities are both an integral component of the rehabilitation process and markers of the relative “normality” of life six months after a stroke, yet, in our findings, the process of adaptation concerning “relearning to eat” varied within the group of participants. For Marie and Anne, their “relearning to eat” was a part of the inpatient neurorehabilitation, while they both had complete independence (FIM score of 7) at discharge. They were ready to go on with their lives while adapting to the other long-term effects of their ABI and did not worry about their severe eating difficulties during hospitalization. For Hans and Peter, it was a little different, because they both had a modified dependence (FIM score of 5) at discharge. They started their process of “relearning to eat” during inpatient neurorehabilitation, but they still needed supervision or a specific facilitation for eating after discharge. They both had to deal with possible losses, as well as a process of adapting to aspects of dependency.

The participants interviewed in the semi-stable phase of rehabilitation [28], nine to 18 months after ABI, stated that they had “normalized” eating, that the meaning of food was the same as before the injury, and that they had reached a point in their rehabilitation process where they did not worry about their eating difficulties.

### 4.3. Eating Meals Together

Peter described being asked to eat in his room with a helper as like being excluded from the patient community. He knew that it was because he needed to concentrate on swallowing and eating and that other people may cause him distraction, but it was still difficult to accept, because he wanted to be part of the social activity surrounding the meals in the ward.

Most of the participants experienced a difficult, but successful, adaptation to daily life over time, regarding eating and drinking with other people during inpatient neurorehabilitation. Considering Spencer’s description [17] of the interactive process of adaptation that occurs between an organism and its environment, and not just on the intrapersonal level [16] to understand our findings, Peter was aware of eating properly when he was part of a dinner at the ward. If something got stuck in his throat and he started coughing then he left the table because he was aware that his coughing should not bother other patients. The participants in this study seemed to have taken, or tried to take, a positive and active role in their own lives.

Similar to our study, Medin et al. [11] found that in striving for the control necessary to eat safely and properly, some individuals avoided activities they would normally perform when eating or participating in something before their stroke. In our study, Peter avoided going out to restaurants or on holidays after discharge. Reasons for not going out as much as usual, with unfamiliar people around, could include fear of coughing and feeling ashamed of not eating properly. Peter’s individual adaptation strategies all served as behavioral adjustments for tackling the following, more complex environmental challenges and to ensure individual survival and self-actualisation [16]. Peter’s new strategies might influence both his and his partner’s social relationships, which might have a long-term negative effect on their quality of life.

### 4.4. Limitations

Due to the small sample of four patients, the conclusions, and generalisation to the ABI population are quite limited. The participants were hospitalised at the same rehabilitation facility and represented a specific group of individuals with ABI [20], with severe eating difficulties at admission, but minor eating difficulties after discharge. The interviews were conducted on a single occasion and the participants had to rely on retrospective recall over considerable time periods. It is also important to consider that the inclusion of participants until saturation might have added additional experiences and findings.

### 4.5. Future Research

Several of the above limitations suggest directions for future research. Future research is needed and it seems that there is more important knowledge of clinical relevance on this topic particularly concerning patients with brain stem infarction starting during the period of inpatient neurorehabilitation in relation to adaptation to the problem and its resolution over time. The important issue in this study was to interview persons with ABI and not proxies, as proxy responses should be used with caution for questions regarding social activities and the degree of satisfaction with participation [36]. It is important to emphasise that all participants were able to verbally articulate their experiences in a reflective and meaningful manner [37]. The optimal future study population would therefore be patients with brain stem infarction and their caregivers. A prospective longitudinal design with a larger sample and with repeated interviews, for example prior to discharge (in the continued rehabilitation phase [28]) and at 6 and 12 months (in the semi-stable phase [28]) and a more long-term follow-up 24 months after discharge, are recommended for future research to explore and interpret how people with ABI and their caregivers experienced and adapted to the problem and its resolution over time. Furthermore, to verify whether direct interventions of information, education and psychological support on this topic can improve patient well-being and make the overall rehabilitation program more effective.

## 5. Conclusions

This preliminary investigation suggests that living with eating difficulties following ABI varies according to individual preferences. The participants reported that they had “normalized” eating. The meaning of food and being able to eat and take part in meals was the same as before the injury; however, having the ability to eat reduced, or lost completely, even temporarily, was experienced as being unexpected and difficult, and caused strong, emotional reactions, even 18 months after the ABI. The time spent using a feeding tube had a negative, but not persistent, impact on quality-of-life. The participants experienced possibilities adapting and developing new strategies for valued activities related to eating as they strived to live their everyday life after injury. Interpretation of the participant experiences in relation to the concept of adaptation contributed insights into the strategies employed by ABI survivors. The findings provide a preliminary step in understanding the patient perspective of adapting and developing new strategies for valued activities related to eating.

## Figures and Tables

**Table 1:** Clinical characteristics of the four participants.

Pseudonym	Marie	Anne	Hans	Peter
Age	18	27	30	60
Sex	Female	Female	Male	Male
Marital status	Single	Married	Single	Married
Diagnosis	Head trauma	Encephalitis	Head trauma	Brain stem infarction
Time since injury (days)	278	526	477	473
Days of inpatient rehabilitation	19	86	129	142
FIM item “Eating” (admission)	1	1	1	1
FIM item (discharge)				
“Eating”	7	7	5	5
“Comprehension”	5	7	5	7
“Expression”	5	7	5	7
“Memory”	5	7	7	7
Type of feeding tube	Nasal	Nasal	Nasal + PEG	Nasal + PEG
Days with feeding tube	31	39	137	172
Days in mechanical ventilation	22	18	26	0
Days of oral intubation	22	14	7	0
Days with tracheostomy tube	0	8	21	0
Aspiration pneumonia	No	No	Yes (acute)	Yes (acute + neurorehabilitation)

FIM, Functional Independence Measure score 1–7 (7 is best). PEG, Percutaneous Endoscopic Gastrostomy.

**Table 2:** Domains in the semi-structured interview guide.

Introduction
1. General questions related to eating and drinking. 2. The meaning of food and liquid before the injury. 3. The meaning of food and liquid at the time of the interview and right after the injury. 4. Do you experience any physical difficulties today, which may influence eating and drinking? What was it like right after the injury? 5. Do you experience worries today and do they influence your mood in relation to eating and drinking? What was it like right after the injury? 6. How is your social life (meals with the family, work, leisure activities, parties, vacations, etc.) today? What was it like right after the injury? 7. What are your experiences of getting food and drink via feeding tube?
**Closing Interview (Debriefing)**

**Table 3:** An example of the codes, sub-categories and a theme from the content analysis of the interviews.

Theme	Eating and Drinking			
Category	Personal Factors	Activity	Body Functions and Structures	
Sub-categories	The meaning of food	Having the first oral intake	Being fed by tube	Food tastes differently
Codes	Meaning of food before and after ABI and at the time of interview Enjoying food before and after ABI and at the time of interview	First food and drink in the mouth after injury	Hunger before and after ABI and at the time of interview Tubefeeding Not being able to eat	Taste of food and drink before and after ABI and at the time of interview

ABI, Acquired Brain Injury.

**Table 4:** An overview of the themes and sub-categories.

**Themes**	3.1. Personal values related to eating	3.2. Swallowing difficulties	3.3. Eating and drinking	3.4. Meals and social life
**Sub-categories**	3.1.1. How to swallow and eat	3.2.1. Symptoms like choking, coughing, voice clearing and salivation	3.3.1. The meaning of food and taking part in meals	3.4.1. Finding a way with my eating difficulties
	3.1.2. Treatment goals concerning swallowing and eating	3.2.2. Worries about swallowing, choking and pneumonia	3.3.2. Being fed by a tube	
		3.2.3. Hunger	3.3.3. Having the first oral intake after ABI	
			3.3.4. Food tastes differently	
